# Six weeks of high-intensity interval training enhances contractile activity induced vascular reactivity and skeletal muscle perfusion in older adults

**DOI:** 10.1007/s11357-021-00463-6

**Published:** 2021-09-25

**Authors:** Philip J. J. Herrod, Philip J. Atherton, Kenneth Smith, John P. Williams, Jonathan N. Lund, Bethan E. Phillips

**Affiliations:** 1 Medical Research Council-Versus Arthritis Centre for Musculoskeletal Ageing Research, University of Nottingham, Royal Derby Hospital Centre, DE22 3DT Derby, UK; 2 NIHR Nottingham Biomedical Research Centre, Queens Medical Centre, Nottingham, UK; 3 Department of Anaesthetics and Surgery, Royal Derby Hospital, Derby, UK

**Keywords:** High-intensity interval training, Ageing, Exercise, Microvascular blood flow, Skeletal muscle

## Abstract

Impairments in muscle microvascular function are associated with the pathogenesis of sarcopenia and cardiovascular disease. High-intensity interval training (HIIT) is an intervention by which a myriad of beneficial skeletal muscle/cardiovascular adaptations have been reported across age, including capillarisation and improved endothelial function. Herein, we hypothesised that HIIT would enhance muscle microvascular blood flow and vascular reactivity to acute contractile activity in older adults, reflecting HIIT-induced vascular remodelling. In a randomised controlled-trial, twenty-five healthy older adults aged 65–85 years (mean BMI 27.0) were randomised to 6-week HIIT or a no-intervention control period of an equal duration. Measures of microvascular responses to a single bout of muscle contractions (i.e. knee extensions) were made in the *m. vastus lateralis* using contrast-enhanced ultrasound during a continuous intravenous infusion of Sonovue™ contrast agent, before and after the intervention period, with concomitant assessments of cardiorespiratory fitness and resting blood pressure. HIIT led to improvements in anaerobic threshold (13.2 ± 3.4 vs. 15.3 ± 3.8 ml/kg/min, P < 0.001), dynamic exercise capacity (145 ± 60 vs. 159 ± 59 W, P < 0.001) and resting (systolic) blood pressure (142 ± 15 vs. 133 ± 11 mmHg, P < 0.01). Notably, HIIT elicited significant increases in microvascular blood flow responses to acute contractile activity (1.8 ± 0.63 vs. 2.3 ± 0.8 (arbitrary contrast units (AU), P < 0.01)), with no change in any of these parameters observed in the control group. Six weeks HIIT improves skeletal muscle microvascular responsiveness to acute contractile activity in the form of active hyperaemia-induced by a single bout of resistance exercise. These findings likely reflect reports of enhanced large vessel distensibility, improved endothelial function, and muscle capillarisation following HIIT. Moreover, our findings illustrate that HIIT may be effective in mitigating deleterious alterations in muscle microvascular mediated aspects of sarcopenia.

## Introduction

Impaired function of the peripheral vasculature becomes increasingly common with advancing age [55], with macro- and microvascular dysfunction often co-existing [54]. Even in the absence of symptomatic peripheral vascular disease, older adults exhibit reductions in whole-limb and microvascular blood flow at rest and in response to exercise, compared to younger adults [15–17]. In terms of the clinical relevance of these deficits in vascular function, it is possible that out with vascular disease per se, they may contribute to the pathogenesis of insulin resistance [32] via impaired delivery of insulin [10, 37], and may also be implicated in the development of hypertension [14]. Another clinical implication of reduced vascular function that has been suggested is its contribution to age-related anabolic blunting and subsequent sarcopenia [67]; the progressive loss of muscle mass and function with advancing age [12]. However, to date, data surrounding this suggestion is equivocal. A number of studies have shown an association between reduced microvascular blood flow and reduced muscle protein synthetic responses to both amino acids [52] and contractile activity [17] — two of the most potent anabolic stimuli [13, 61], in older adults. Conversely, other research, including that by our group has suggested that neither macro- nor microvascular blood flow responsiveness is rate-limiting for muscle protein anabolism based on studies of exercise [48], pharmacological [50] and nutritional enhancement [49] of vascular responses to nutrition. Irrespective, enhanced vascular function offers potential benefit for muscle, cardiovascular and metabolic health [18, 51].

Despite the reported benefits of both lifelong exercise and exercise uptake on numerous aspects of physiological function, including central (i.e., cardiovascular [9]) and peripheral vascular function [56], low adherence to ‘traditional’, often time-consuming exercise interventions such as those described in common public health guidelines [45], [66] is a limitation to achieving these benefits. In older adults, adherence to this type of exercise training is consistently described as poor, with rates as low as 10% reported [30, 38, 43]. The rationale for this lack of engagement in older adults is largely akin to those reported across all age-groups, with a perceived lack of time at the fore [6, 39]. Additionally, already poor physical health is also a reported factor in this age-group [42, 44]. High-intensity interval training (HIIT) is an alternative training mode to traditional moderate intensity continuous training (MICT) which may potentially engender increased uptake and adherence due to its time-efficiency (i.e., shorter session duration [33]) and ability to quickly improve factors pertaining to cardiorespiratory health and fitness in both young [4] and older [25] adults. Supporting this notion, a systematic review and meta-analysis of 65 studies concluded that HIIT has the ability to improve cardiorespiratory fitness over both short- (< 12–weeks) and longer-term training durations [2]. Although not all studies have demonstrated superiority of HIIT when compared to MICT [19, 21], in terms of absolute improvements in cardiorespiratory fitness, its time-efficient nature is unquestionable [24].

To date, little is known about the effect of HIIT on limb blood flow in older adults and we are unaware of any studies of microvascular blood flow in response to HIIT in any age group. Studies have shown improvement in popliteal artery endothelial function following HIIT in both older and younger adults [46], whilst HIIT in young adults has also been shown to increase expression of endothelial nitric oxide synthase [11]. However, none have examined the effect of HIIT on microvascular blood flow.

Therefore, the primary aim of this study was to assess the effect of 6 weeks’ time-efficient HIIT on microvascular responsiveness in healthy older adults.

## Methods

### Subject characteristics

Ethical approval (A12092016) was obtained from the University of Nottingham Faculty of Medicine and Health Science Research Ethics Committee to recruit adults aged 65–85 years to participate in a trial of 6-week fully supervised HIIT versus a non-intervention control. All participants gave written informed consent to participate. Exclusion criteria included current participation in a formal exercise regime, a BMI < 18 or > 30 kg·m^2^, active cardiovascular disease, uncontrolled hypertension (> 160/100 mmHg), diabetes mellitus, family history of early (< 55 years) death from cardiovascular disease or known sensitivity to Sonovue™ contrast agent. The study was registered with clinicaltrials.gov and complied with the 1964 Declaration of Helsinki. Patients were randomised via sealedenvelope.com, using random permuted blocks.

Before baseline testing, all subjects underwent a screening session including a cardiovascular examination by a qualified medical doctor with additional exclusion criteria for further participation as per the American Thoracic Society (ATS)/American College of Chest Physicians (ACCP) guidelines for Cardiopulmonary Exercise Testing (CPET) [64]. A unilateral one-repetition maximum (1-RM) assessment for knee extension was also conducted at this screening session [36] to set the intensity of the RE to be used as our vasodilatory stimulus. At baseline testing, participants underwent measures of skeletal muscle (*m. vastus* lateralis) microvascular responses to RE by contrast enhanced ultrasound (CEUS) and completed a CPET and resting blood pressure (BP) assessment, as per our previously published protocols [26]. After the 6-week intervention period, all baseline tests were repeated (~ 72 h after the final HIIT session). Participants were requested to maintain their habitual dietary intake for the duration of the study and to consume a standardised evening meal prior to an overnight fast (> 10 h, water ad libitum) before each testing session.

### Microvascular blood flow

Using our standard CEUS methods as previously described [41], skeletal muscle microvascular blood flow (MBF) in the *m. vastus lateralis* was measured at rest and in response to a single bout of unilateral RE. In brief, CEUS measures were made using a contrast-enabled ultrasound machine (Philips iU22, Phillips Healthcare, Guildford, UK) and a 9–3-mHz probe housed in a custom-made probe-holder secured to the leg with Velcro straps. The probe was secured at mid-thigh level based on anatomic reference to the inguinal crease and mid-point of the patella. The probe was attached to the participant’s thigh for 20 min before CEUS measurements to allow the probe temperature to equilibrate with that of the participant’s skin [57] Sonovue™ (Bracco, Milan, Italy) was used as the microbubble ultrasound contrast agent and was prepared and administered (intravenously into an antecubital fossa vein) as per manufacturer’s instructions.

With participants seated on an isometric leg extension machine (ISO Leg extension, Leisure Lines (GB) Ltd., Hinckley, UK), and after a measure of resting BP [25], a single vial of Sonovue™ was infused at a rate of 2 ml/min for 1 min before reduction to 1 ml/min until the syringe was empty. Each vial of Sonovue™ provided ~ 4 min of infusion time, allowing 90 s of infusion to achieve systemic steady state [41] and a further 2.5 min to assess MBF.

**Fig. 1:**
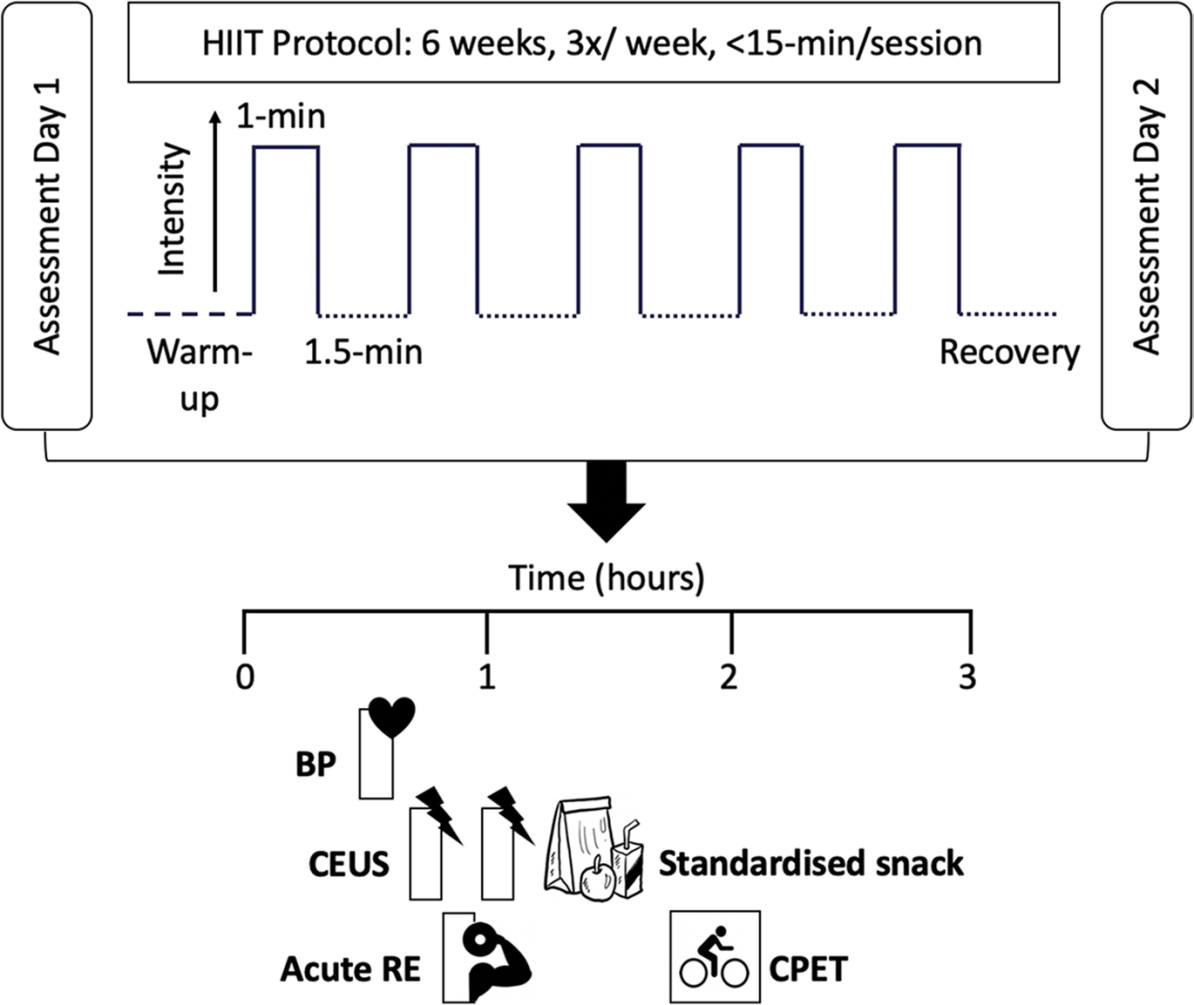
Schematic representation of study protocol, including assessment days (right) and high intensity interval training (HIIT) (left). Abbreviations: CEUS, contrast enhanced ultrasound; RE, resistance exercise; CPET, cardiopulmonary exercise testing

To determine MBF, microbubbles under the probe which had accumulated during the first 90 s were destroyed using a brief “flash” impulse of high mechanical index ultrasound from the probe [41]. This flash and the replenishment of contrast agent to the muscle underlying the probe was recorded on two subsequent 30-s cine clips through the probe. Participants were then instructed to perform 6 unilateral isotonic knee extensions at 50% 1-RM [36]. After this, participants were asked to keep their legs completely still and another set of flash-replenishment cycles were recorded to determine skeletal muscle microvascular responsiveness to the RE bout (Fig. 1).

DICOM video files of the flash-replenishment cycles were analysed using QLAB image quantification software (Version 10, Phillips Healthcare) as previously described [41]. Regions of interest (ROI) were drawn freehand including as much muscle as possible, whilst excluding connective tissue or any large rapid filling vessels (distinct large vessels visibly containing contrast agent within the first 0.05 s of post-flash recording) and were copied across all cine clips for each participants’ visit to ensure consistency of measurements. QLAB then provided a value for the mean acoustic index (AI) (a measure of the echogenicity of the tissue, which increases proportionately to the concentration of the Sonovue™ [41]) in the ROI for each frame of the cine clip. The mean AI for the first 6 frames immediately post-flash was designated the background AI of the tissue within the ROI and subtracted from all subsequent AI values.

The mean AI data from the pre-RE cine clips were averaged to give one measure of pre-exercise microvascular blood flow which was compared to the mean post-exercise AI data. AI values from both before and after RE were then used to plot curves of AI against time, and determine the exponential function of one-phase association using:

$$\mathbf{Y}={\mathbf{Y}}_{0}+\mathbf{A}\left(1-{\mathbf{e}}^{-{\varvec{\upbeta}}\mathbf{t}}\right),$$

where A denotes the level of the plateau of the graph and is analogous to MBF (volume) within the tissue ROI [57].

### Cardiopulmonary exercise testing

CPET was performed according to ATS/ACCP guidelines [64] using a Lode Corival cycle ergometer (Lode Corival, Lode, Groningen) and inline gas analysis system (ZAN 680, nSpire Health, Colorado, USA) as previously reported [25]. In brief, after 2 min of unloaded cycling, participants were instructed to maintain a cadence of 50–60 revolutions per minute whilst being encouraged to exercise to volitional exhaustion. A Bruce ramp protocol [34, 65] was selected (10–20 W per minute) based on the participant’s body weight and self-reported level of habitual physical activity to ensure the CPET was between 8 and 12 min in duration [8, 64]. Anaerobic threshold (AT) was determined using a combination of the V-slope and VE methods [3, 62] by two blinded independent assessors, with disagreement resolved by consensus. As the same ramp protocol was employed per participant for both baseline and post-intervention (or control period) assessments, the maximum wattage achieved during CPET was considered representative of dynamic exercise capacity.

### High-intensity interval training (HIIT)

Subjects assigned to HIIT attended the laboratory three times each week for 6 weeks, with each session lasting approximately 15 min (including a warm-up, 5, 1-min high intensity cycling efforts and a recovery period). The HIIT regime has been described previously [5, 47]. All training sessions were fully supervised and conducted with 12-lead ECG, BP and pulse oximetry safety monitoring. After 3 weeks of HIIT (at the end of the 9^th^ training session), participants were asked to rate their perceived exertion on modified Borg scale of 1–10 [7]. A rating of ≤ 8 led to a 10% increase in training intensity for the remaining HIIT sessions [47]. Subjects in the control group attended for pre and post intervention testing sessions only. Compliance for the HIIT sessions was 100%, with all participants attending all of their allocated sessions.

### Statistics

All calculations were performed using GraphPad Prism Version 9.0 (California, USA). Data are presented as mean (SD). Participant demographics at baseline were compared using an unpaired t-test, whilst outcome data were compared using two-way ANOVA (group × time). Significance was accepted as an alpha of p < 0.05. Effect size is reported as Cohen’s *d*.

## Results

### Participant characteristics

Twenty-five participants were recruited and randomly allocated to either HIIT or the no-intervention control. In one participant assigned to the HIIT group, measurement of MBF was not achieved due to cannula failure leading to contrast extravasation. One participant allocated to the control group did not return for the post intervention visit and was lost to follow-up. Therefore, 23 participants were included in the final analysis. No physiological parameter was different between the two groups at baseline (Table 1).

**Table 1:** Participant characteristics, microvascular blood volume, anaerobic threshold and exercise capacity at baseline

	HIIT (*n* = 13)	CON (*n* = 12)
Age (y)	70 (3)	72 (6)
Sex (male/female)	5/8	7/5
BMI (kg/m^2^)	27.7 (2.4)	26.3 (2.9)
AT (ml/kg/min)	13.2 (3.4)	15.1 (6.0)
W_max_	145 (60)	144 (63)
SBP	142 (15)	130 (10)
DBP	85 (13)	80 (10)
MBV responsiveness	1.8 (0.63)	2.2 (1.2)

Data are presented as mean (SD). Abbreviations: *BMI*, body mass index; *AT*, anaerobic threshold; *W*_*max*_, maximum wattage achieved during cardiopulmonary exercise testing; *SBP*, systolic blood pressure; *DBP*, diastolic blood pressure; *MBV*, microvascular blood volume (change in response to a single-set of isotonic knee extension exercise)

### Anaerobic threshold (AT)

**Fig. 2:**
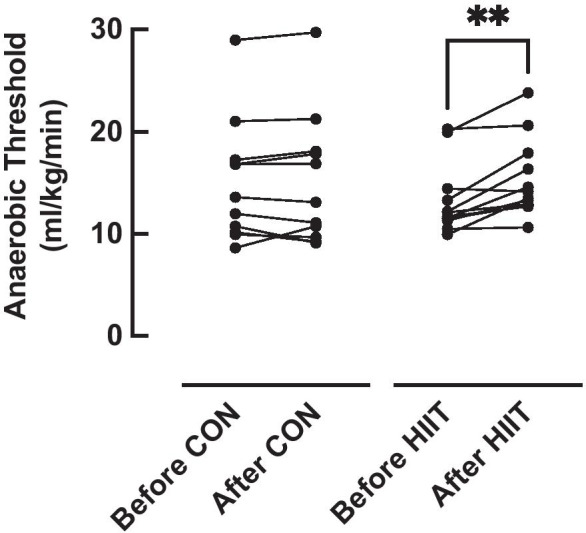
Anaerobic threshold (AT) before and after 6-week high intensity interval training (HIIT, *n* = 13) or an equivalent no-intervention control period (CON, *n* = 12). Analysis via two-way ANOVA. ** = *p* < 0.01

There was no significant difference in baseline AT between groups (*P* = 0.37) (Table 1). There was however a significant effect of time (*P* < 0.01) and a significant group × time interaction (*P* < 0.01), with post-hoc testing demonstrating a significant increase in AT after the intervention period in the HIIT group only (13.2 (3.4) vs. 15.3 (3.8) ml/kg/min, *P* < 0.01; effect size (ES): 0.56), with no change in the control group (15.1 (6.0) vs. 15.2 (6.4) ml/kg/min, *P* = 0.98; ES: 0.01) (Fig. 2).

### Exercise capacity

Used as an indicator of dynamic exercise capacity, there was no significant difference in baseline peak wattage achieved during the CPET (W_peak_) between groups at baseline (*P* = 0.96) (Table 1). There was a significant effect of time (*P* < 0.01) and a significant group × time interaction (*P* < 0.01), with post-hoc testing demonstrating a significant increase in W_peak_ after the intervention period in the HIIT group only (145 (60) vs. 159 (59) W, *P* < 0.01; ES: 0.25), with no change in the control group (144 (63) vs. 145 (62) W, *P* = 0.95; ES: 0.01).

### Blood pressure (BP)

**Fig. 3:**
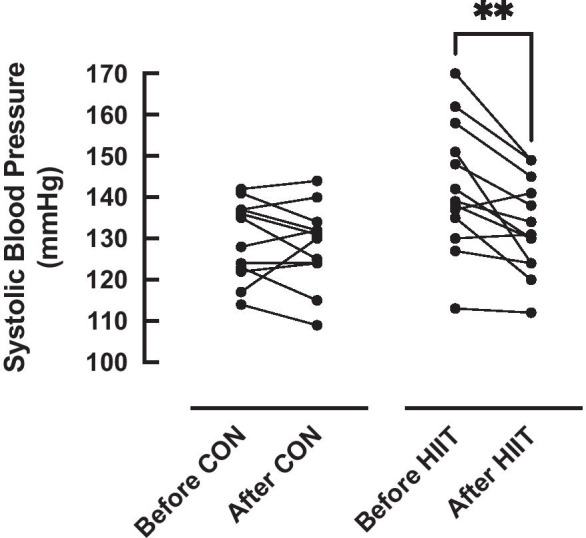
Systolic blood pressure (SBP) before and after 6-week high intensity interval training (HIIT, *n* = 13) or an equivalent no-intervention control period (CON, *n* = 12). Analysis via two-way ANOVA. ** = *p* < 0.01

There was no significant difference in resting systolic BP (SBP) between the groups at baseline (*P* = 0.78) (Table 1). There was a significant effect of time (*P* < 0.01) and a significant group × time interaction (*P* = *0*.02), with post-hoc testing demonstrating a significant reduction in SBP after the intervention period in the HIIT group only (142 (16) vs. 133 (11) mmHg, *P* < 0.01; ES: − 0.67), with no change in the control group (130 (10) vs. 128 (10) mmHg, *P* = 0.81; ES: − 0.05) (Fig. 3). There was no significant difference in diastolic blood pressure (DBP) between the groups at baseline (*P* = 0.78) (Table 1), nor was there a significant effect of time (*P* = 0.27) on DBP, or group × time interaction (*P* = *0*.76). The coefficient of variation (CV) for repeated SBP assessments within our laboratory is 2.57%.

### Microvascular blood volume (MBV)

**Fig. 4:**
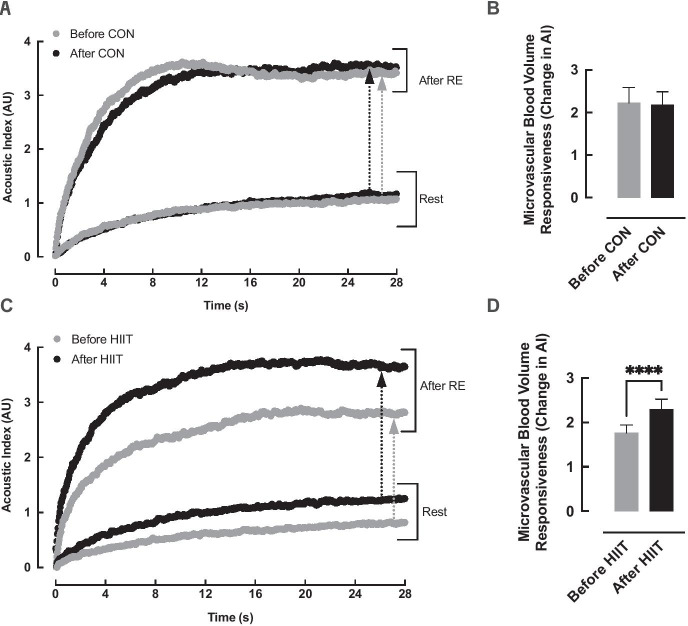
Microvascular blood volume (MBV) responses to an acute bout of resistance exercise (RE; knee extensions) before and after 6-week high intensity interval training (HIIT (**C/D**), *n* = 13) or an equivalent no-intervention control period (CON (**A/B**), *n* = 12). Panels **A** and C show microvascular refilling curves before and after the HIIT (**C**) or control (**A**) period with arrows representing the increase in MBV (as increase in acoustic intensity (AI)) in response to RE before (grey) and after (black) the HIIT or CON period. Analysis via two-way ANOVA. ** = p < 0.01

There was no significant difference in MBV responses to a single set of 6 unilateral knee extensions between the groups at baseline (*P* = 0.24) (Table 1). There was a significant effect of time (*P* = 0.02) and a significant group × time interaction (*P* < 0.01), with post-hoc testing demonstrating a significant increase in MBV responses to RE after the intervention period in the HIIT group only (1.8 (0.6) vs. 2.3 (0.8), *P* < 0.01; ES: 0.64), with no change in the control group (2.2 (1.2) vs. 2.2 (1.0), *P* = 0.94; ES: − 0.12) (Fig. 4). The CV for repeated MBV assessments within our laboratory is 1.09%.

**Fig. 5:**
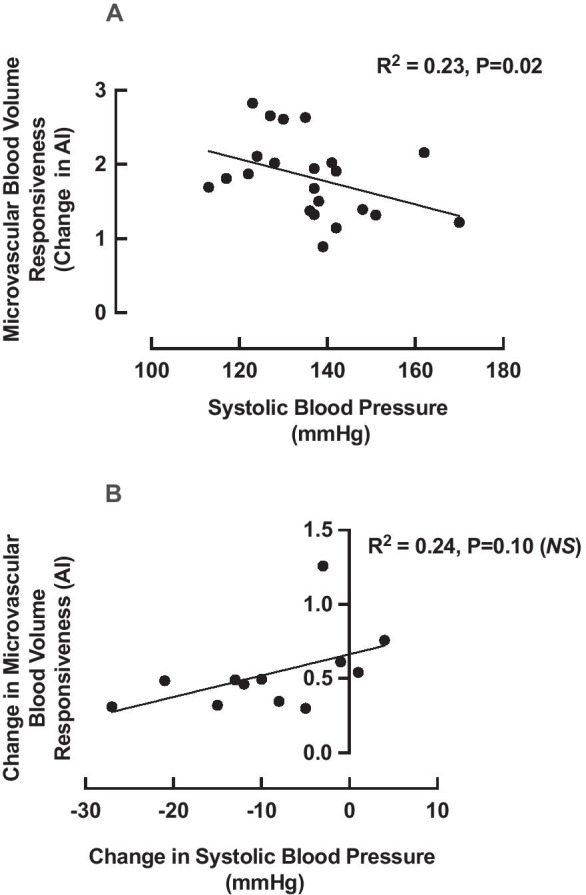
The relationship between (**A**) systolic blood pressure (SBP) and microvascular responsiveness (as increase in acoustic intensity (AI)) at baseline (*n* = 25) and (**B**) change in SBP with high intensity interval training and change in microvascular responsiveness (*n* = 13). Analysis via Pearson’s correlation. *NS*: non-significant

In assessing the relationship between microvascular blood flow responsiveness and blood pressure, we observed a significant relationship (when the two groups were combined) between baseline SBP and microvascular blood flow responsiveness (change elicited by the bout of RE) (R^2^ = 0.23; *P* = 0.02 (Fig. 5A)), a relationship that was not apparent for DBP (R^2^ = 0.01; *P* = 0.60) or mean arterial pressure (R^2^ = 0.01; *P* = 0.18). Further, based on the reduction in SBP that was observed with HIIT, we sought to explore the relationship between changes in SBP with HIIT, and changes in MBF responsiveness elicited by HIIT. There was no relationship between these changes, (R^2^ = 0.24; *P* = 0.10 (Fig. 5B).

## Discussion

This work demonstrates that 6 weeks, time-efficient HIIT on a cycle ergometer performed 3 times each week elicits improvements in the CRF and SBP of older adults. Further, this HIIT protocol enhanced MBF responsiveness to an acute bout of RE in the functionally important *vastus lateralis* muscle [60]. To our knowledge, this is the first study to evaluate changes in MBF in response to HIIT, in any age group. We believe these microvascular changes may elicit favourable adaptation to concomitant resistance exercise training, given the known association between vascular dysfunction and skeletal muscle mass/function [18] and as such the effects of multimodal training with both HIIT and RET in the same muscle merit further study.

The underlying reason behind improvements in MBF responsiveness following HIIT may be due to increased capillarization of the muscle [31]. However, previous work comparing the MBF of young and older men with measurements of muscle capillarization via muscle biopsies has shown an age-related decline in MBF, despite no corresponding reduction in capillarization [28], implying that age-related impairment in this regard is functional rather than structural. This does not preclude increased capillarization being a mechanism behind our observed increases in MBF responsiveness following HIIT, as angiogenesis has been demonstrated in young [29] and older [23] adults following exercise training, including HIIT [1]. This assertion may also be supported by a previous observational study of 23 healthy middle-aged adults which performed resting CEUS measurements of MBV in the *vastus lateralis*, before taking biopsies of the same muscle for histological analysis. In this study, MBV significantly correlated with the number of capillary-muscle fibre contacts [63]. In addition, even if increased capillarization is a contributory mechanism for our observed increase in MBV after HIIT, increased capillarization may only be a local effect at the quadriceps as this is the muscle most used in cycle ergometer training [58]. As such, the ability for HIIT to influence aspects of muscle microvascular blood flow in other muscles or indeed tissues (remote to the main contraction site) remains to be tested. Other potential explanations for our HIIT-induced increase in MBV responsiveness, outside of increased capillarisation, include improved vasodilation at the level of the resistance arterioles [59]. Supportive of this suggestion, six-week HIIT has previously been shown to increase the expression of endothelial nitric oxide synthase (the enzyme responsible for the production of the majority of nitric oxide, a potent vasodilator [20]) in young men [11], but there is no data to support this proposition in older adults.

Of note, this study is one of the few to use CEUS to measure muscle microvascular blood flow responsiveness before and after a chronic exercise intervention. Whilst this has been done in both the upper [53] and lower limb [48] in response to RET, this is the first to measure the response following chronic HIIT. Previous chronic training studies using CEUS to measure muscle MBV have, like us, only compared the change in microvascular responsiveness to a stimuli (e.g. feeding) [48] due to CEUS assessment of baseline MBF parameters over a chronic time-period not yet being validated. As such, although we are not able to report on changes in baseline MBF with HIIT, it is interesting that the baseline rested/fasted MBF of our control group (in the same location based on anatomic reference points) was not different when comparing before and after the intervention period (p = 0.31), whilst an increase was observed after HIIT (p = 0.03). The primary concerns regarding the chronic application of CEUS is ensuring the same region of the muscle is studied before and after an intervention period, as even if the probe position is marked based on static anatomical landmarks (i.e., the mid-point of the patella), changes in the underlying tissue composition (i.e., due to hypertrophy and/or adipose losses) would alter the measurement region. Baseline to baseline comparisons have been reported in one study; however, this study made no comment as to the validity of this comparison and lacked any non-intervention control group to demonstrate repeatability of measurements [53]. As such, although our findings are suggestive of HIIT-induced improvements in basal MBF in older adults, further work is needed to validate CEUS for this application.

That SBP at baseline was associated with pre-intervention MBF responsiveness, likely represents improved endothelial function in those with more desirable blood pressure at baseline [27]; however, there was no association between MBF and pre-intervention DBP or MAP and MBF. Despite the correlation between baseline SBP and pre-intervention MBF responsiveness, and HIIT leading to significant improvements in both MBF and SBP, there was no correlation between the changes in both parameters. This may be due to the presence of both normotensive and hypertensive individuals in this study, with normotensive individuals perhaps being unable to (or indeed needing to) elicit a significant reduction in SBP whilst still making improvements in MBF responsiveness.

In addition, although the clinical significance of HIIT-induced increases in MBV responses to RE has yet to be determined, it may have positive effects on concomitant resistance exercise training, offering a potential strategy to combat sarcopenia and the numerous detrimental consequences of this condition [35]. That the improved responsiveness was observed 72 h after the final HIIT session, seemingly negates the need for HIIT and RET to perform in close temporal proximity (i.e., in the same session or even on the same day), removing concerns related to session length, fatigue and ‘interference’ during concurrent exercise [22], and potentially supports a mixed-modality training regime for older adults where HIIT can not only improve CRF and SBP as demonstrated herein, but may also potentially be able to augment RET-induced gains.

One limitation of this study is that it recruited only healthy older adults, excluding those with conditions known to impact vascular function such as obesity and diabetes. As such, these results may not be replicated in patient groups with these conditions. However, it may be that those with sub-optimal physiological function can achieve enhanced benefits from our HIIT protocol, as has been seen elsewhere in the literature [40]. This limitation is apparent in a large number of studies exploring physiological parameters/mechanisms, which often employ stringent inclusion/exclusion criteria to reduce participant heterogeneity. Whilst these studies are important to explore new paradigms, we fully support the need for studies of this nature to subsequently be conducted in clinical/at-risk population groups, who are potentially more likely to benefit from these findings.

## Abbreviations

AI: Acoustic index
CEUS: Contrast enhanced ultrasound
CPET: Cardiopulmonary exercise test
HIIT: High-intensity interval training
MBF: Microvascular blood flow
MBV: Microvascular blood volume
MFV: Microvascular flow velocity
MICT: Moderate-intensity continuous training
RE: Resistance exercise
